# In Silico and In Vitro Characterization of *Bacillus velezensis* P45: Screening for a Novel Probiotic Candidate

**DOI:** 10.3390/foods14132334

**Published:** 2025-06-30

**Authors:** Carolini Esmeriz da Rosa, Cristian Mauricio Barreto Pinilla, Luiza Dalpiccoli Toss, Adriano Brandelli

**Affiliations:** 1 Laboratory of Nanobiotechnology and Applied Microbiology, Department of Food Science, Federal University of Rio Grande do Sul, Porto Alegre 91501-970, Brazil; caroliniesrosa@gmail.com (C.E.d.R.); luizatoss@hotmail.com (L.D.T.); 2 Dairy Technology Center, Institute of Food Technology, Campinas 13083-015, Brazil; cristian@ital.sp.gov.br

**Keywords:** antimicrobial peptides, *Bacillus*, genome analysis, gut adhesion, probiotics

## Abstract

Spore-forming *Bacilli* have been explored due to their potential biotechnological features and applications in human health and functional food research. This study focuses on the genetic and phenotypical characterization of the functional probiotic properties of *Bacillus velezensis* P45, a strain isolated from fish intestines. *B. velezensis* P45 exhibited antimicrobial activity against Gram-positive and Gram-negative pathogens and demonstrated strong autoaggregation and biofilm formation properties in vitro. The strain also showed tolerance to gastrointestinal conditions and ability to metabolize and adhere to mucin. In silico analysis confirmed the absence of virulence factors and antibiotic resistance genes, reinforcing its safety as a probiotic candidate. Genome mining revealed the presence of genes related to adhesion, such as fibronectin-binding protein and enolases, and for the synthesis of secondary metabolites, including the antimicrobial lipopeptides fengycin, surfactin, and bacillibactin. In addition, phylogenetic comparison using the *yloA* (*rqcH*) gene associated with gut adhesion clustered strain P45 with other probiotic *Bacillus* and *B. velezensis* strains, while separating it from pathogenic bacteria. Thus, the strain *B. velezensis* P45 could be a valuable candidate as a probiotic due to its functional properties and safety.

## 1. Introduction

The interest in probiotics has grown exponentially in recent decades, driven by the increasing consumer demand for products associated with health benefits. Probiotics can promote intestinal homeostasis, stimulating the immune system [1,2]. Currently, it has been well established that the influence of probiotics extends beyond the gastrointestinal tract [3]. These microorganisms can contribute to the regulation of physiological processes in distant organs, including the brain, skin, lungs, and cardiovascular system, by engaging complex immune, metabolic, and neural pathways [4,5]. In addition, substances derived from microbial metabolism or cell-inactivated forms (postbiotics) have been widely studied due to their impact on the dynamics of probiotic action in promoting health benefits to the host [6,7]. Examples of postbiotics include antimicrobial peptides and short-chain fatty acids (SCFAs) that typically exhibit inhibitory activity against undesirable bacterial species and anti-inflammatory activities, respectively [8,9]. The selection criteria for probiotic microorganisms, as outlined by the World Health Organization (WHO), the Food and Agriculture Organization of the United Nations (FAO), and the European Food Safety Authority (EFSA), encompass requirements related to their safety, functional capabilities, and beneficial properties [10]. The desirable properties include the capability to survive and maintain metabolic activity under gastrointestinal tract (GIT) conditions, the absence of genes associated with antimicrobial resistance, and the ability to adhere and colonize in the host [10,11].

Among the genera traditionally and commercially recognized for their probiotic potential, *Lacticaseibacillus* is one of the most extensively studied bacterial groups [12]. Strains of this genus have been extensively used due to its strong ability to adhere to intestinal epithelial cells, tolerate gastric acidity, modulate the host immune response, and reduce gastrointestinal disorders in both children and adults [13,14,15]. Its safety profile and documented health benefits have led to its wide application in functional foods and clinical research [16].

In comparison, *Bacillus* strains have been less represented in probiotic formulations; however, increasing evidence supports their potential in human and animal use [17,18,19]. *Bacillus* species such *B. subtilis*, *B. paralicheniformis*, and *B. velezensis* and their postbiotics have received their safety status from various international regulatory agencies, such as GRAS (Generally Recognized As Safe) by the U.S. FDA and QPS (Qualified Presumption of Safety) by the EFSA, indicating that these microorganisms have been evaluated and are considered safe for use based on scientific evidence and regulatory assessment [20,21,22].

Additionally, the spore-forming capacity in the probiotic field is limited, although the inherent resistance of spores provides a distinct advantage over other genera since this feature can ensure high survival rates during industrial processing and storage, as well as enhanced resistance to the harsh conditions encountered in whole gastrointestinal tract of humans and/or animals [23,24]. Once established in the intestinal environment, *Bacillus* strains can exert health-promoting effects on the host, through the production of diverse functional secondary metabolites and digestive enzymes [18,25]. These include ribosomally and non-ribosomally synthesized antimicrobial peptides, polyketides, and lipopeptides such as surfactin, fengycin, and iturin [24].

Thus, the properties of *Bacillus* species, including *B. velezensis*, can contribute to the potential probiotic effect by modulating host immune responses, playing a key role in competitive exclusion and host–microbe interactions [26]. These functional effects have also been associated with clinical benefits, such as alleviating gastrointestinal symptoms in individuals with inflammatory bowel syndrome [27,28]. *B. velezensis* strains have been adopted as safe probiotic agents in animal feed and aquaculture and some works shown their potential as human and animal probiotics [29,30,31].

*Bacillus velezensis* P45, isolated from the gut of a fish native to the Brazilian Amazon basin, has shown promising probiotic properties. This strain has demonstrated an ability to produce bioactive compounds such as antimicrobial lipopetides and exhibits antimicrobial activity against a range of bacteria [32]. Considering the increasing interest for novel probiotic strains, there is limited information about probiotic *B. velezensis*, particularly on the use of genome-wide approaches. This study aims to evaluate the properties of *B. velezensis* P45 through genome screening to evaluate the presence of genetic elements encoding *Bacillaceae*-probiotic properties, secondary metabolites, virulence factors, and antibiotic resistance. In vitro assays were used to further understand antibiotic resistance and probiotic features to assess the potential of strain P45 as a probiotic for health and nutrition applications.

## 2. Materials and Methods

### 2.1. Strains and Culture Conditions

*Bacillus velezensis* P45, previously isolated from the fish *Piaractus mesopotamicus* [33], along with other bacterial strains used in this study, were retrieved from the culture collection of the Laboratory of Biochemistry and Applied Microbiology (UFRGS, Porto Alegre, Brazil) and conserved at −20 °C in Brain-Heart Infusion broth (BHI; Kasvi, Pinhais, PR, Brazil) containing 20% (*v*/*v*) glycerol. The strains used in this study were *Listeria monocytogenes* ATCC 7644, *Clostridium perfringens* ATCC 3624, *Enterococcus faecalis* LAB-4, *Salmonella enterica* sv. Enteritidis ATCC 13076, *Pseudomonas aeruginosa* 4B, *Proteus vulgaris* IC-1, and *Staphylococcus aureus* ATCC 25923. These strains were cultivated in BHI agar at 37 °C for 24 h. *Lacticaseibacillus rhamnosus* CCT 7863, provided by Gabbia Biotecnologia (Itajái, SC, Brazil), was cultured on De Man, Rogosa, and Sharpe (MRS; Merck, Darmstadt, Germany) agar under anaerobic conditions by 48 h at 37 °C, prior the tests.

### 2.2. Safety Assessment

#### 2.2.1. Hemolytic Activity Assay

*B. velezensis* P45 was inoculated onto blood agar plates (Laborclin, Pinhais, PR, Brazil) and incubated for 24 h at 37 °C. Hemolytic reaction was classified based on the appearance of partial hydrolysis as a yellow zone around the colonies (α-hemolysis), a clear zone around colonies (β-hemolysis), or no reaction (γ-hemolysis) [34]. *S. aureus* ATCC 25923 was used as a positive control.

#### 2.2.2. Antibiotic Susceptibility Test

Antimicrobial resistance of *B. velezensis* P45 was evaluated using disc diffusion method against the following 15 antimicrobials (Cefar, São Paulo, Brazil): amikacin (30 μg/disc), ampicillin (10 μg/disc), chloramphenicol (30 μg/disc), ciprofloxacin (5 μg/disc), clindamycin (2 μg/disc), erythromycin (15 μg/disc), gentamicin (10 μg/disc), imipenem (10 μg/disc), levofloxacin (5 μg/disc), linezolid (30 μg/disc), meropemem (10 μg/disc), norfloxacin (10 μg/disc), penicillin (10 μg/disc), tetracycline (30 μg/disc), and vancomycin (30 μg/disc). The strain was cultured in BHI plates and incubated at 37 °C for 24 h and then diluted in saline solution (0.85% NaCl) standardized to OD_600_ of 0.5. The bacterial solution was then inoculated on Mueller Hinton (Kasvi) agar plates. The antibiotic discs were manually placed on the plate surface and incubated at 37 °C for 18 h. The results were expressed considering the inhibition zone diameter (mm) and categorized as resistant (R), with moderate susceptibility (MS), or susceptible (S), based on the parameters of the Clinical and Laboratory Standards Institute (CLSI) [35]. The experiment was performed as three biological replicates.

### 2.3. Evaluation of Probiotic Properties

#### 2.3.1. Antagonistic Activity

The antibacterial activity was evaluated by the agar diffusion test [36]. The antagonism activity of the *B. velezensis* P45 was tested against *L. monocytogenes*, *E. faecalis*, *C. perfringens*, *S.* Enteritidis, *P. aeruginosa*, and *P. vulgaris*. Test microorganisms were prepared in a concentration of 10^8^ CFU/mL in saline solution (0.85% NaCl, *w*/*v*) and were swab-inoculated onto BHI agar plates surface. Serial dilutions of the crude supernatant from *B. velezensis* P45 cultures were grown in BHI broth at 37 °C for 24 h and then added to fresh lawn cultures. The plates were then incubated aerobically at the optimal temperature for each test microorganism. *C. perfringens* was incubated under anaerobic conditions at 37 °C. Clear zones of growth inhibition, indicating positive antagonism activity, were measured. The antagonistic activity was quantified in activity units (AU) per mL, defined as the reciprocal of the highest dilution that produces a clear zone of inhibition [37].

#### 2.3.2. Autoaggregation and Cell Surface Hydrophobicity

Aggregation ability of *B. velezensis* P45 was assessed as described previously [38], with minor modifications. *L. monocytogenes* ATCC 7644 and *L. rhamnosus* CCT 7863 were used as references for pathogenic and probiotic bacteria. Briefly, *B. velezensis* P45 and *L. monocytogenes* were cultured in BHI broth at 37 °C for 24 h, while *L. rhamnosus* was grown in MRS broth. The cultures were harvested via centrifugation at 6000× *g* for 10 min at 4 °C, and the pellets were washed twice with PBS (10 mM Na_2_HPO_4_, 2 mM NaH_2_PO_4_, 150 mM NaCl, pH 7.4). The bacterial pellets were then suspended in PBS and incubated at 37 °C for 24 h. During incubation, the optical density (OD600) of the upper fraction of the suspensions was determined at 0, 2, 4, 6, and 24 h. The autoaggregation rate was calculated using the following equation:

Autoaggregation (%) = 1 − (*A*t/*A*_0_) × 100 (1)

where *A*t is the absorbance at t2, t4, t6, and t24, and *A*_0_ is the initial absorbance.

Cell surface hydrophobicity of *B. velezensis* P45, *L. monocytogenes* ATCC 7644, and *L. rhamnosus* CCT 7863 were evaluated using microbial adhesion to hydrocarbons [39]. The strains were prepared as described above. After suspension in PBS, 3 mL of bacterial cells were mixed with 400 μL of dichloromethane or xylene and vortexed for 60 s. The following equation was used to calculate the bacterial affinity to hydrocarbons:

Hydrophobicity (%) = (*H*_0_ − *H*t)/*H*_0_ × 100 (2)

where *H*t is the absorbance values measured at 600 nm after the incubation period, and *H*_0_ is the recorded at the start of the experiment.

#### 2.3.3. Biofilm Formation Assay

Biofilm formation was assessed under static conditions using a previously established method [40], with minor adjustments. Briefly, an overnight culture of *B. velezensis* P45 was inoculated (1%) into wells of a 96-well polystyrene microplate, each containing 300 μL BHI or BHI prepared with 0.1 µg/mL ampicillin, which served as a negative control. The microtiter plates were incubated for 6 and 24 h at 37 °C. Biofilm formation was evaluated according to the crystal violet (CV) method [41].

#### 2.3.4. Tolerance to Gastrointestinal Conditions

The tolerance of *B. velezensis* P45, to in vitro gastrointestinal conditions was determined as described elsewhere [42], with minor modifications. *L. monocytogenes* ATCC 7644 and *L. rhamnosus* CCT 7863 were included for comparison. The bacterial strains were prepared as described above and then suspended in simulated gastric acid (SGA: 125 mM NaCl, 7 mM KCl, 45 mM NaHCO_3_, 3 g/L pepsin, pH 2.0) and incubated at 37 °C for 90 min. After this period, the suspensions were centrifuged at 6000× *g* for 10 min, the pellets were washed with PBS, and then suspended in simulated intestinal fluid (SIF: 22 mM NaCl, 3.2 mM KCl, 7.6 mM NaHCO_3_, 0.1% pancreatin and 0.15% bile salts, pH 8.0) for 150 min at 37 °C. At the end of each stage of the analysis, aliquots were collected, and the bacterial counts were measured using serial dilutions in BHI for *B. velezensis* and *L. monocytogens* and MRS for *L. rhamnosus*.

#### 2.3.5. Bacterial Adhesion to Mucin

The ability of *B. velezensis* P45 to adhere to the mucin layer was assessed as described previously [43], with some modifications. *L. monocytogenes* ATCC 7644 and *L. rhamnosus* CCT 7863 were included for comparison. For the test, type-II porcine gastric mucin (Sigma Aldrich, St. Louis, MO, USA) was prepared in PBS (10 mg/mL) and immobilized in a 96-well polystyrene microplate through overnight storage at 4 °C. After washing the microplate twice with PBS, a bovine serum albumin (BSA) solution (20 mg/mL) was added to each well for a saturation step, which was carried out for 4 h at 4 °C. Each strain was cultivated in their respective optimal media and at their ideal temperatures. All cultures were centrifuged at 10,000× *g* for 15 min, and bacterial cells were suspended in PBS to achieve approximately 10^8^ CFU/mL. A volume of 100 µL of each strain suspension was added to the wells and incubated 1.5 h at 37 °C. The supernatants were removed, and the plates were washed twice with PBS to remove unbound bacteria. Adhering bacteria were desorbed with 0.25% trypsin/EDTA solution (Corning Inc., Corning, NY, USA) for 2 min. Finally, 100 µL samples from each well were plated on BHI or MRS agar plates to determine viable cell counts. Prior to the assay, the trypsin-EDTA solution used in the analysis was evaluated on all strains to prevent any adverse effect on bacterial viability.

#### 2.3.6. Mucin Growth Assay

The growth ability of *B. velezensis* P45 in the presence of type-II porcine gastric mucin was assessed and compared with *L. monocytogenes* ATCC 7644 and *L. rhamnosus* CCT 7863. Overnight cultures of *B. velezensis* P45 and *L. monocytogenes* were inoculated (1%) into BHI broth, while *L. rhamnosus* was inoculated into MRS broth, both containing 1 mg/mL of type-II porcine gastric mucin. The respective media culture without mucin served as the control. Samples were incubated at 37 °C for 12 h and the optical density (OD600) was measured every 1 h interval.

### 2.4. Whole Genome Analysis

DNA extraction, sequencing, and alignment of *B. velezensis* P45 have been reported previously [32]. This Genome Shotgun project is deposited at EMBL/ENA/GenBank under accession code: JAFJZY000000000.1. Probiotic features within the *B. velezensis* P45 genome were explored through manual analysis of coding DNA sequences using the online platform Rapid Annotation using Subsystem Technology (RAST) and SEED v.2.0 (http://rast.nmpdr.org (accessed on 15 February 2025)). The analysis focused on identifying protein-derived genes related to potential probiotic functions, such as adhesion, aggregation, vitamin biosynthesis, amino acid metabolism, and adaptations to the host gastrointestinal tract. Additionally, gene clusters associated with the biosynthesis of antimicrobial compounds were identifying using the antiSMASH 7.0 tool [44]. A detailed examination of genes with an over-85% identity to reference sequences was performed using Blast KOALA (KEGG Orthology and Links Annotation), and the characterization of biosynthetic gene clusters with PRISM4 (http://prism.adapsyn.com (accessed on 23 March 2025)) [45] was performed for identification of non-ribosomal peptides, type-I and -II polyketides, and RiPPs. BAGEL4 web server was used to identify gene clusters involved in the biosynthesis of ribosomally synthesized and post-translationally modified peptides (RiPPs) and (unmodified) bacteriocins [46].

Furthermore, the genome sequence of the strain *B. velezensis* P45 was submitted to the iProbiotics database [47] for an assessment of its probiotic potential. iProbiotics employs *k*-mer frequencies to characterize complete bacterial genomes and uses the support vector machine algorithm for probiotic identification.

### 2.5. Alignment and Phylogenetic Reconstruction

Aiming to illustrate the presence and arrangement of adherence genes, we reconstruct a phylogenetic analysis tree created with *yloA* (*rqcH*) adhesion gene. For the phylogenetic analysis, we run BLASTn with the gene *rqcH* coding sequence (CDS); then, the amino acids sequences were aligned using MUSCLE, implemented within MEGA version 11.0 software package [48]. To manage variation and exon counts among taxa, we trimmed the alignment using Gblocks 0.91.1 with specific parameters: smaller final blocks and gap positions within the blocks [49]. The phylogenetic analysis was conducted using maximum likelihood in IQTree 1.6.12 [50]. Tree topology confidence values were evaluated with 10,000 bootstrap replicates, and the results were visualized using iTOL 6.8.1 [51].

### 2.6. Statistical Analysis

Statistical analysis was conducted using GraphPad Prism version 8.0.1, employing statistical tests suitable for each type of analysis. Data are presented as the mean values ± standard error of the mean, with a threshold for statistical significance set at *p* < 0.05. All experiments were conducted in triplicate.

## 3. Results

### 3.1. Safety Assessment

*Bacillus velezensis* P45 was tested for susceptibility to various antimicrobials and exhibited sensitivity to all tested agents (Table S1). The strain P45 exhibited γ-hemolysis, showing no hemolytic zone, while *S. aureus* ATCC 25923, used as the positive control, demonstrated β-hemolytic activity. The lack of hemolytic activity and susceptibility to 17 commonly used antimicrobials are significant safety attributes, suggesting that *B. velezensis* P45 is a safe candidate for use as a probiotic.

### 3.2. Probiotic Properties

#### 3.2.1. Antimicrobial Activity

**Figure 1:**
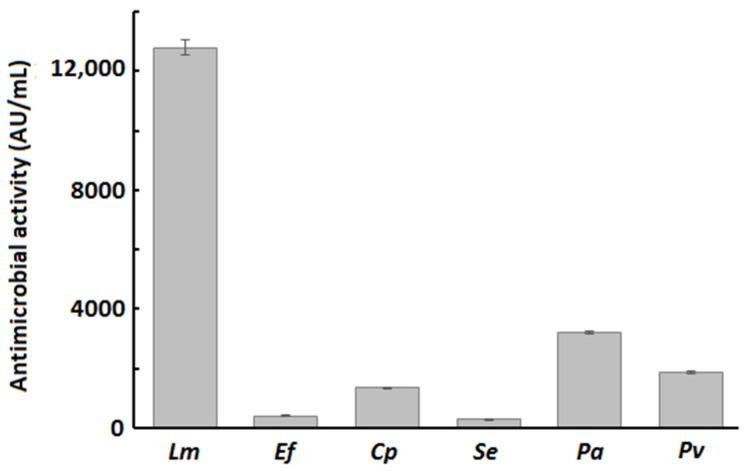
Antimicrobial activity of crude supernatant of B. velezensis P45 against *Listeria monocytogenes* (Lm), *Enterococcus faecalis* (Ef), *Clostridium perfringens* (Cp), *Salmonella enterica* (Se), *Pseudomonas aeruginosa* (Pa), and *Proteus vulgaris* (Pv). Values are mean ± SEM from three biological replicates.

The antibacterial activity of *B. velezensis* P45 was evaluated against different bacterial species. The crude culture supernatant of P45 showed antagonistic activity against the strains tested (Figure 1). Specifically, the highest antimicrobial activity was recorded against *L. monocytogenes*, while lower values were observed for other bacteria.

#### 3.2.2. Autoaggregation and Cell Surface Hydrophobicity

**Figure 2:**
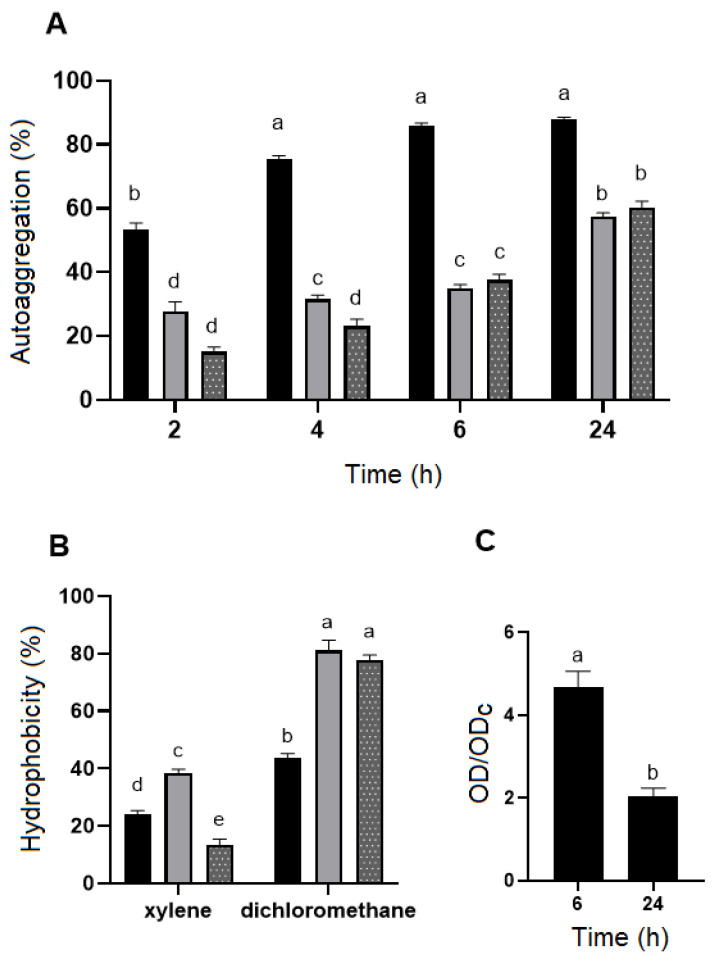
(**A**) The autoaggregation ability (%) and (**B**) surface hydrophobicity (%) of *Bacillus velezensis* P45 (black bars), *Listeria monocytogenes* ATCC 7644 (grey bars), and *Lacticaseibacillus rhamnosus* CCT 7863 (dotted bars) in different times and with different organic solvents, respectively. (**C**) Biofilm formation by *Bacillus velezensis* P45. Values are expressed as the mean ± SEM from three biological replicates. Different letters indicate significant differences (*p* < 0.001).

*B. velezensis* P45 demonstrated a higher autoaggregation rate among the strains, with a percentage that increased over time, reaching around 88% at 24 h (Figure 2A). In comparison, *L. rhamnosus* CCT 7863, used as the control, showed a maximum autoaggregation of 60% at 24 h, while *L. monocytogenes* ATCC 7644 exhibited the lowest autoaggregation rate among the strains at 57% (Figure 2A).

Cell surface hydrophobicity was evaluated using xylene (a non-polar solvent) and dichloromethane (a polar solvent). All strains demonstrated moderate-to-high hydrophobicity towards dichloromethane, with *B. velezensis* P45, *L. rhamnosus* CCT 7863, and *L. monocytogenes* ATCC 7644 showing hydrophobicity values of 43.9%, 81.2%, and 77.3%, respectively (Figure 2B). However, hydrophobicity values with xylene were lower, with *B. velezensis* P45 at 24.2%, *L. rhamnosus* CCT 7863 at 38.3%, and *L. monocytogenes* ATCC 7644 at 13.3%.

#### 3.2.3. Biofilm Formation

Biofilm formation was assessed using the CV assay, which is commonly applied to quantify total biofilm formation on a specific synthetic surface. The data indicated that the biofilm formation of *B. velezensis* P45 was considered strong during the first 6 h but decreased over time (Figure 2C).

The interpretation of results was based on the scale proposed by Stepanović et al. (2007) [41]. Thus, the average optical density values were calculated for each well stained with crystal violet, measured at 570 nm. A cut-off value (ODc) was established, defined as three standard deviations (SD) above the mean OD of the negative control. The ODc serves as a reference point to determinate the significance of biofilm formation, with levels of CV staining above this threshold indicating notable biofilm presence or adherence.

#### 3.2.4. Tolerance to Gastrointestinal Conditions

**Figure 3:**
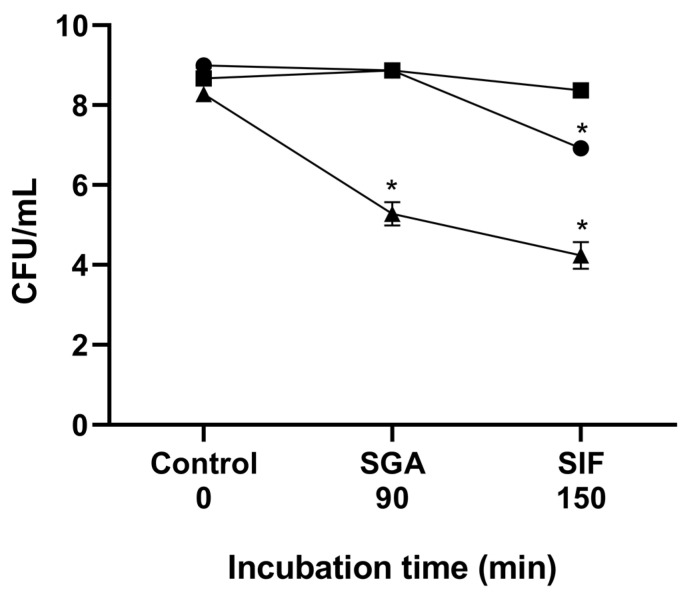
Cell viability during gastrointestinal simulation in vitro. The lines show cell viability before simulation, after exposure to simulated gastric acid (SGA), and after simulated intestinal fluid (SIF). (●) *Bacillus velezensis* P45, (■) *Lacticaseibacillus rhamnosus* CCT 7863, and (▲) *Listeria monocytogenes* ATCC 7644. (*) Statistical differences (*p* < 0.001) are indicated by an asterisk.

The tolerance of *B. velezensis* P45, *L. monocytogenes* ATCC 7644, and *L. rhamnosus* CCT 7863 to gastrointestinal conditions was determined in vitro to assess cell viability upon exposure to simulated gastric acid and intestinal fluids. All strains were initially introduced into the simulation at a concentration of approximately 10^8^ CFU/mL. As a result, *L. rhamnosus* CCT 7863 showed a high survival rate post-simulation, suggesting tolerance to these conditions (Figure 3). *B. velezensis* P45 and *L. monocytogenes* ATCC 7644 showed decreased cell viability following exposure to simulated fluids. Viable counts of *B. velezensis* decreased of approximately 1.5 log CFU/mL, but only after SIF treatment. In contrast, *L. monocytogenes* ATCC 7644 exhibited a reduction of 4 log CFU, suggesting that the gastrointestinal conditions had a significant impact on the survival of this pathogen.

#### 3.2.5. Adhesion and Growth in Mucin

**Figure 4:**
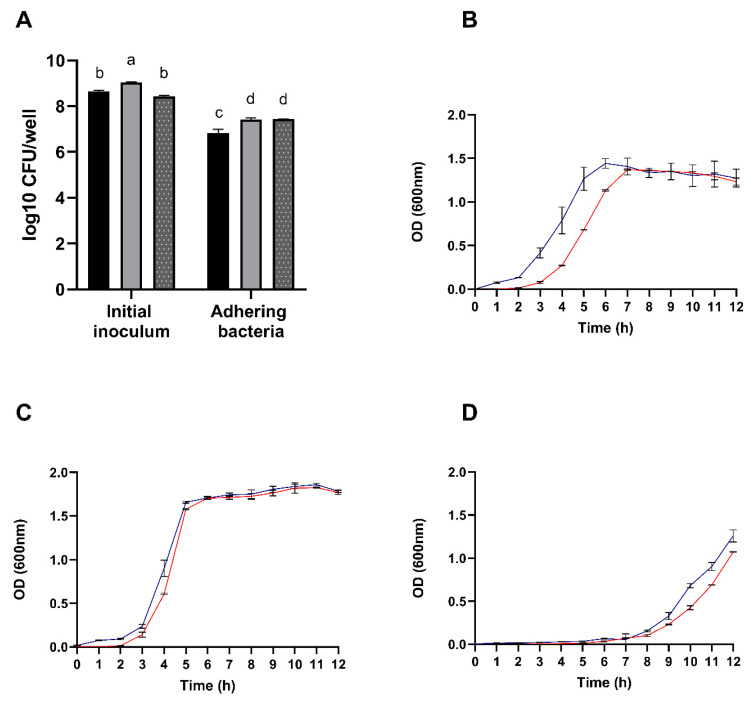
(**A**) Enumeration of adhering *Bacillus velezensis* P45 (black bars), *Listeria monocytogenes* ATCC 7644 (grey bars), and *Lacticaseibacillus rhamnosus* CCT 7863 (dotted bars) cells to mucin. Growth curves of *Bacillus velezensis* P45 (**B**), *Lacticaseibacillus rhamnosus* CCT 7863 (**C**), and *Listeria monocytogenes* ATCC 7644 (**D**) in the presence (blue line) or absence (red line) of porcine mucin. Values are expressed as the mean ± SEM from three biological replicates. Different letters denote significant differences.

The overall mucin adhesion capabilities of the species under study is summarized in Figure 4A. Results revealed adhesion percentages ranging from 79% to *B. velezensis* P45, 88% to *L. rhamnosus* CCT 7863, and 82% for *L. monocytogenes* ATCC 7644.

Interestingly, as shown in the Figure 4B, the growth of *B. velezensis* P45 was positively influenced by the presence of porcine mucin, suggesting that the strain can thrive on mucin as a substrate. In contrast, *L. rhamnosus* CCT 7863 and *L. monocytogenes* ATCC 7644 exhibited similar growth patterns regardless of mucin presence. Therefore, the combined adhesion and growth results suggest that *B. velezensis* P45 has a potential capacity to effectively colonize the host intestinal mucosa.

### 3.3. Genome Mining for Probiotic Characteristics

The genome annotation of *B. velezensis* P45 using RAST predicted several putative genes associated with probiotic traits (Table S2). These characteristics include adhesion and aggregation, vitamin biosynthesis, amino acid metabolism, lactic acid production, active metabolism, enzyme production for food digestion, and tolerance to stress and the host gastrointestinal tract. Moreover, the probiotic prediction was performed on the iProbiotics web server, revealing that strain P45 could be confirmed (99.88%) as a probiotic (Figure S1).

#### 3.3.1. Genes Related to Virulence and Antimicrobial Resistance

Bioinformatics tools were employed to investigate the presence of genes related to virulence and antibiotic resistance in the *B. velezensis* P45 genome. As result, no chromosomally encoded antibiotic resistance genes were found. Additionally, analysis with the PlasmidFinder web tool revealed the absence of plasmids in *B. velezensis* P45. This absence of plasmids is a favorable probiotic indicative as plasmids can harbor genes for antimicrobial resistance and virulence factors. Analysis using VFanalyzer from the Virulence Factor Database found no matches, indicating the absence of virulence factors. The PathogenFinder web server was also used to assess potential pathogenicity towards human health, predicting *B. velezensis* P45 as non-pathogenic for humans. The probability of being a human pathogen was calculated as 0.109, with no matches to pathogenic families and 56 matches to non-pathogenic families.

#### 3.3.2. Genes Related to Antimicrobial Metabolites

AntiSMASH analysis was employed to predict the biosynthetic gene clusters for secondary metabolites potentially associated with antimicrobial activities. The genome of *B. velezensis* P45 revealed six secondary metabolite gene clusters. Those exhibiting over 80% similarity to known compounds were specifically noted. The biosynthesis machinery and bioactivities of these clusters are detailed in Table 1.

Tree gene clusters related to nonribosomal peptide synthetases (NRPS) related to the synthesis of cyclic lipopeptides (CLPs) were identified. Clusters 2 (fengycin), 3 (surfactin), and 6 (bacillibactin) exhibited 80, 95, and 100% similarity with *B. velezensis* FZB42 gene clusters, respectively. Clusters 4 and 5 were categorized as polyketide synthases (PKS). Cluster 4 is associated with the synthesis of bacillaene, whereas Cluster 5 is related to the production of macrolactin H. Both clusters showed 100% similarity with PKS clusters of *B. velezensis* FZB42 gene clusters. Cluster 1 was classified as “Other” and is associated with the synthesis of bacilysin, showing 100% similarity with known clusters.

**Figure 5:**
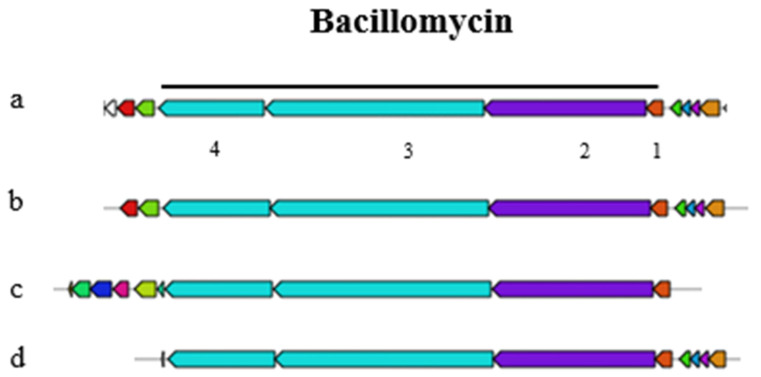
Bacillomycin gene cluster of the query sequence *Bacillus velezensis* P45 (**a**) with the core biosynthetic genes constituted by (1) MCAT (2) *bmyA*, (3) *bmyB*, and (4) *bmyC* under the black line; (**b**) bacillomycin; (**c**) mycosubtilin and gene clusters with 100% similarity; and (**d**) an iturin cluster with 88% similarity.

A representation of the fengycin gene cluster is provided in Figure S2, highlighting the key genes involved in the synthesis of this antifungal peptide and its relationship with plipastatin. Interestingly, the bacillomycin gene cluster, which is part of the iturin family, is not an independent entity but is integrated within the fengycin gene cluster (Figure 5). This arrangement suggests a complex genetic architecture where multiple biosynthetic pathways are co-located. The genomic region responsible for siderophore biosynthesis was found to be an exact match to that of bacillibactin. Likewise, the genomic region governing the synthesis of the tail-cyclized peptide was identified as a precise duplicate of amylocyclicin (Figure S3).

Analysis of *B. velezensis* P45 using the NP.searcher program revealed the presence of mixed modular NRPS/PKS, trans-ATPKs, and a non-mevalonate terpenoid *mep* gene. Additionally, BAGEL4 analysis identified six distinct bacteriocins and RiPP clusters associated with the production of antimicrobial peptides and bacteriocins (Table S3). Clusters linked to antimicrobial activity include sactipeptides, amylocyclicin, UviB, and LCI. Further structural predictions were made using the PRISM4 algorithm, which indicated 12 clusters of putative compounds, including seven NRPs, four PKs, one Class II/III confident bacteriocin (thiopeptide), one bacterial head-to-tail cyclized peptide, and one ComX (Table S4).

### 3.4. Phylogenetic Comparison of yloA (rqcH) Adhesion Gene

**Figure 6:**
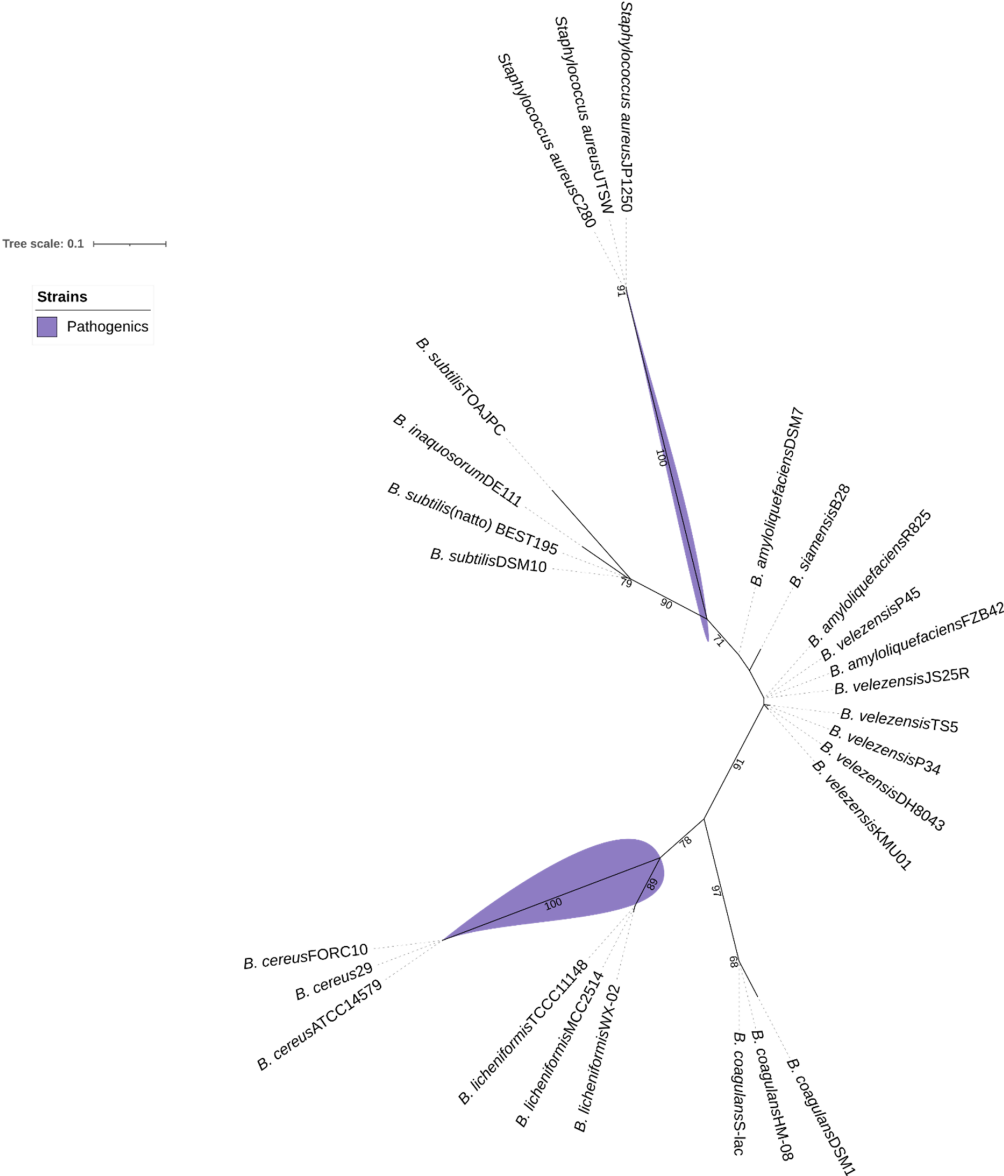
Maximum-likelihood tree based on gene *rqcH* sequences, showing relationships between *Bacillus velezensis* P45, probiotic *Bacillus*, and some pathogenic bacteria. Bootstrap values are shown on branches.

The *yloA* (or *rqcH*) gene of interest, associated with adhesion, was selected for comparative analysis based on whole-genome alignments of the examined *Bacillus* strains. This analysis indicates the presence of a tendency for probiotic and non-probiotic *Bacillus* strains to cluster separately from typically pathogenic *Bacillus* species, such as *Bacillus cereus*, and from other genera, such as the *Staphylococcus* group. As shown in Figure 6, *B. velezensis* P45 clustered with strains of probiotic bacilli and was separated from the pathogenic strains, thereby supporting its potential as a probiotic. Several strains classified as probiotics or potential probiotics were grouped with strain P45, including *B. velezensis* TS5, *B. velezensis* KMU01, *B. amyloliquefaciens* R825, *B. siamensis* B28, and *B. velezensis* FZB42. In contrast, pathogenic and non-probiotics groups clustered separated, including *B. cereus* ATCC 14579, *B. cereus* FORC10, *B. cereus* 29, *B. amyloliquefaciens* DSM7, and *B. licheniformis* TCCC11148.

## 4. Discussion

The increasing demand for preventive healthcare, combined with the demonstrated efficacy of probiotic bacteria, has driven significant interest in probiotics and their metabolites in recent decades [52]. Despite the genus *Bacillus* not being abundantly found in commercial formulations, recent advances on the probiotic potential of *Bacillus* strains have been achieved [19,23,24,25,26,30]. To address this issue, the strain *B. velezensis* P45, isolated from the intestine of an Amazonian fish, was evaluated in the present study.

The strain P45 exhibited no hemolysis, as reported for probiotic *Bacillus* strains [11,53], but also in *B. velezensis* candidates [30,54], suggesting that the absence of hemolytic activity is a consistent property across *B. velezensis* strains. Moreover, the antimicrobial susceptibility test revealed that the strain P45 is widely susceptible to all antibiotics tested. In silico genomic analyses of *B. velezensis* P45, utilizing PlasmidFinder, VFanalyzer, and PathogenFinder platforms, confirmed these findings. No genetic sequences related to virulence factors (VF), transferable antimicrobial resistance (AMR), or mobile genetic elements were identified in the genome of strain P45. Although resistance to certain antibiotics is frequently observed in *Bacillus* species exposed to environmental stresses associated to agricultural practices and animal production [55,56], lower resistance was noted in strains like *B. velezensis* P45, which was isolated from an environment with minimal antibiotic exposure. These findings suggest that P45 is a potentially safe strain for probiotic use, aligning with the broader trend of promising probiotic candidates.

*B. velezensis* is generally recognized as safe (GRAS) bacterium, which has been extensively used in agriculture and as feed additive with potential health benefits [29,57]. In this regard, it could be expected that *B. velezensis* P45 is also a probiotic strain, as predicted with a very high score (99.88%) by the iProbiotics platform. The iProbiotics prediction is achieved by using machine-learning algorithms to analyze the presence of certain *k*-mers, which are short and contiguous nucleotide sequences in the genome. The iProbiotics tool has been trained on a large dataset of 239 known probiotic strains (41 species) and 412 non-probiotic strains (80 species), supporting accurate predictions based on the presence or absence of specific *k*-mers [47]. The score obtained by *B. velezensis* P45 was higher than those of other *Bacillus* also proposed as probiotic candidates [58].

The antagonistic activity of *B. velezensis* P45 against pathogenic strains demonstrated significant antimicrobial effect, with particularly higher activity against *L. monocytogenes*. This finding corroborates previous studies showing that *B. velezensis* P45 produces bacillomycin D and fengycins A and B in BHI medium [32]. These antimicrobial lipopeptides are recognized by their broad antimicrobial activity, including Gram-positive and Gram-negative bacteria [24,31,57]. Moreover, *Bacillus subtilis* and *Bacillus cereus* strains isolated from fish intestines exhibited antimicrobial activity against *L. monocytogenes* and *Salmonella* [59]. Similarly, lipopeptides from *Bacillus* sp. P34, also isolated from fish intestines, effectively inhibited *L. monocytogenes*, *S. aureus* [60], and food isolates of *S. aureus* and *E. faecalis* [61]. Furthermore, *B. subtilis* KATMIRA1933 and *B. amyloliquefaciens* B-1895 inhibited biofilm formation by various *Salmonella* serovars through the production of subtilosin A and subtilin, known antibiotic peptides produced by *Bacillus* species [62]. These findings are consistent with the antimicrobial activity of *B. velezensis* P45 against pathogenic bacteria, suggesting that *Bacillus* strains share common metabolites and mechanisms for pathogen inhibition. This reinforces the value of *Bacillus* strains with probiotic properties, not only to improve intestinal health but also to control pathogens in food systems.

Genome analysis of strain P45 revealed gene clusters encoding antimicrobial lipopeptides such as fengycin, surfactin, iturin bacillaene, macrolactin, and amylocyclin. These compounds are synthesized by *Bacillus* species through large enzymatic complexes known as non-ribosomal peptide synthetases (NRPS) [63]. Notably, the bacillomycin gene cluster, part of the iturin family, was not identified as an independent cluster but rather integrated within the fengycin gene cluster in the genome of P45, which could be due to the close physical proximity of the plipastatin/fengycin and iturin genes on the chromosome [64]. Furthermore, gene clusters within a contig may not meet antiSMASH criteria for forming distinct genetic clusters, which could result in the incomplete detection of clusters, particularly in fragmented genomes [65]. Moreover, gene clusters involved in the biosynthesis of two polyketides (macrolactin H and bacillaene) and one peptide (bacilysin) were also identified, with 100% similarity, consistent with data from *Bacillus* sp. P34 [60]. The gene cluster for bacillibactin, a typical siderophore of the *Bacillus* genus, plays a crucial role in iron acquisition, plant growth promotion, and antibiotic activity [66,67].

Furthermore, the adhesion capacity to the intestinal epithelium plays a critical role in the efficacy of probiotic strains as it directly affects various aspects of the host microbiota. Adhesion increases the residence time of probiotics in the gut, facilitating colonization and promoting the establishment of beneficial microbiota communities [68]. By occupying adhesion sites, probiotics inhibit the attachment of pathogens, thereby preventing colonization and mitigating potential adverse effects [69,70]. Autoaggregation and hydrophobicity index are common indicators of the adhesion ability of bacterial strains. In comparison to other strains used in this study, namely, *L. rhamnosus* CCT 7863 and *L. monocytogenes* ATCC 7644, *B. velezensis* P45 exhibited superior autoaggregation rates at all time points, reaching 55% at 2 h and the maximum value of 88% at 24 h. These elevated rates were also higher as compared to other *Bacillus* species. For example, *Bacillus paralicheniformis* FA6 achieved an autoaggregation rate of 28.4% after 2 h in PBS [71], while *Bacillus* sp. RCS1 reached 80.2% after 24 h [72]. The autoaggregation rate of *B. velezensis* P45 also exceeded the results reported for well-known probiotics like *L. rhamnosus* GG, which displayed a rate of 59–65% [73,74], and *Bifidobacterium pseudolongum* YY-26, which demonstrated a rate of 68.9% [75].

*Bacillus velezensis* P45 exhibited strong biofilm formation during the early hours of incubation, followed by a notable decline after 24 h. Several environmental, cellular, and surface-related factors can directly affect bacterial adhesion and biofilm development [76,77]. Although the crystal violet assay is widely recognized and applied for evaluating biofilm formation in *Bacillus* species, we acknowledge that specific regulatory features or physiological responses of the strain P45 may have influenced the dynamics observed under our experimental conditions. In particular, the limited medium volume (300 μL) may have contributed to nutrient depletion and metabolite accumulation, potentially triggering a reduction in biofilm mass after 24 h. This nutrient stress could also initiate sporulation process, thereby reducing the number of vegetative cells and, consequently, the retention of crystal violet [77,78].

The mucus layer on the gastrointestinal epithelium serves as a primary barrier against pathogenic microorganisms and as a substrate that supports the commensal bacteria, facilitating their growth, biofilms formation, and intestinal colonization [43,79]. Porcine gastric mucin is commonly employed as a model to simulate human mucin, allowing for the investigation of bacterial adhesion and providing valuable insights into microbial interactions with the gastrointestinal epithelium [80,81]. This model provides valuable insights into how mucins affect the adhesive capabilities or growth of the microorganisms of interest. Both *B. velezensis* P45 and *L. rhamnosus* CCT 7863 demonstrated enhanced growth in the presence of porcine mucin. Similarly, several *Lacticaseibacillus plantarum* strains isolated from fermented foods showed increased growth with mucin, indicating the ability to utilize it as a growth substrate [81].

Tolerance to gastrointestinal tract conditions is another essential ability for probiotic strains [82]. Simulation of gastrointestinal tract conditions provides a reliable method for assessing probiotic behavior. In this study, *B. velezensis* P45 and the probiotic strain *L. rhamnosus* CCT 7863 exhibited tolerance to gastric and intestinal fluids, whereas *L. monocytogenes* ATCC 7944 demonstrated a significant decrease in cell viability. Adaptation to the gastrointestinal conditions can be mediated by the expression of genes encoding for proteins like arginine/ornithine antiporter, F_0_F_1_-ATP synthase, Na^+^/H^+^ antiporter, nhaX and nhaK antiporters, and sodium bile acid symporter, maintaining the homeostasis in the gastrointestinal tract [83,84]. In this context, genome analysis of *B. velezensis* P45 revealed the presence of protein-coding sequences (CDS) related to reduce bacterial damage. Over 40 genes involved in tolerance to adverse gastrointestinal conditions were identified, including resistance to low pH, high bile salt concentrations, oxidative and osmotic stress, and temperature tolerance (Table S2). Genes related to DNA repair were also identified, such as the nuclease ABC complex (uvrA, uvrB, uvrC), which further enhance the strain resilience. The presence of genes encoding for the arginine/ornithine antiporter ArcD, ornithine carbamoyl transferase, arginine pathway regulatory protein ArgR, F_0_F_1_-ATP synthase subunits, L-lactate dehydrogenase, Na^+^/H^+^ antiporter, and bile acid sodium symporter reinforce the tolerance of strain P45 to acidic conditions and high bile salt concentrations [83,84,85]. In addition, endospores formation, a characteristic of the *Bacillus* genus, enables these microorganisms to withstand various environmental stresses [77,84].

Based on genome information, *B. velezensis* P45 harbors over 15 genes putatively associated with adhesion-related proteins, including chitin-binding protein, sortase D, fibronectin-binding protein, enolases, and flagellar hook-basal body complex proteins (Table S2), which supports adhesion properties of *B. velezensis* P45. These results are consistent with other studies that discovered similar genes in potential probiotic *Bacillus*, such as *B. velezensis* ZBG17 [86], *B. velezensis* FS26 [31], and *B. cereus* G1-11 [87].

Finally, the biosynthesis of vitamins, amino acids, and cofactors by probiotic bacteria underscores their ability to produce essential bioactive compounds. Since the human body is not capable of synthesizing all the required vitamins, their intake is essential for several biological processes, such regulating biochemical reactions and maintaining homeostasis by participating in metabolic pathways as precursors of intracellular coenzymes [88]. In *B. velezensis* P45, genes involved in the synthesis of water-soluble vitamins were identified, including B2 (riboflavin), B7 (biotin), B1 (thiamine), B3 (niacin), B6 (pyridoxine), B5 (pantothenic acid), B6 (pyridoxine), B9 (folic acid), and B12 (cobalamin). Additionally, genes coding for the biosynthesis of fat-soluble vitamins, such as vitamin K (menaquinone), were also detected. Thus, incorporating probiotic bacteria into food products may provide a natural source of essential vitamins, directly synthesized in the digestive tract, modulating the abundance and diversity of gut microbiota promoting intestinal and overall health [88,89].

## 5. Conclusions

This study highlights the potential of *B. velezensis* P45 as a promising probiotic candidate. Several in vitro assays demonstrate its strong adhesion capability, its tolerance to stress conditions (including acid, bile, osmotic, and oxidative stress), and its ability to synthesize secondary metabolites such as vitamins and antimicrobial compounds, corroborated by in silico identification of several genes encoding these properties. These attributes are related to various metabolic and biochemical processes that help maintain viability in GIT and inhibit the growth of pathogens. Both in vitro and in silico analyses indicate that *B. velezensis* P45 synthesizes antimicrobial compounds, which are essential for competitive exclusion of pathogens and for its survival in the host gut. Additionally, the strain exhibits a favorable safety profile, with no evidence of antibiotic resistance, virulence factors, or pathogenicity. These findings underscore the potential of *B. velezensis* P45 as a probiotic candidate for human health, favoring the further exploration of strains of the *B. velezensis* genus for applications in the food and pharmacy sectors. However, further in vivo studies are necessary to confirm the probiotic potential and functional effects of *B. velezensis* P45.

## Figures and Tables

**Table 1:** Gene clusters found in *Bacillus velezensis* P45 genome using antiSMASH software considering synthesis pathways, the most similar known gene clusters, the bioactivity, and the similarity with *Bacillus velezensis* FZB42 gene clusters.

Cluster Type ^1^	Most Similar Cluster	Bioactivity	Similarity (%)
1. Other	Bacilysin	Antibacterial	100
2. NRPS	Fengycin	Antifungal	86
3. NRPS	Surfactin	Surfactant	95
4. PKS-NRPS	Bacillaene	Antibacterial	100
5. PKS	Macrolactin H	Antibacterial	100
6. NRPS	Bacillibactin	Siderophore	100

^1^ NRPS, non-ribosomal peptide synthase; PKS, polyketide synthase.

## Data Availability

The genome of *B. velezensis* P45 is available at EMBL/ENA/GenBank under the accession code: JAFJZY000000000.1. The original contributions presented in this study are included in the article/Supplementary Materials. Further inquiries can be directed to the corresponding author.

## Supplementary Materials

The following supporting information can be downloaded at https://www.mdpi.com/article/10.3390/foods14132334/s1: Table S1: *Bacillus velezensis* P45 susceptibility to antimicrobials by disc diffusion assay; Table S2: Genes related to potential probiotic characteristics in *Bacillus velezensis* P45 genome; Figure S1: Probability prediction of *B. velezensis* P45 genome as probiotics using the iProbiotics algorithm; Figure S2: Biosynthetic gene cluster for fengycin of the query sequence *Bacillus velezensis* P45; Figure S3: Bacillibactin gene cluster of the query sequence *Bacillus velezensis* P45 identified by antiSMASH 7.0; Table S3: Predicted bacterial RiPP clusters in *Bacillus velezensis* P45 genome based on BAGEL4; Table S4: Predicted secondary metabolites gene clusters in *Bacillus velezensis* P45 genome based on PRISM4.
