# Supplementary material for: In Silico and In Vitro Characterization of Bacillus velezensis P45: Screening for a Novel Probiotic Candidate
Source: Foods. 2025 Jun 30;14(13):2334. doi: 10.3390/foods14132334 (PMC12249262; doi:10.3390/foods14132334)
Supplement: Supplementary file 1 [file foods-14-02334-s001.zip › foods-3695589-supplementary.pdf]

foods

Supplementary material

In silico and *in vitro* characterization of *Bacillus velezensis* P45: screening for a novel probiotic candidate

Carolini Esmeriz da Rosa, Cristian Mauricio Barreto Pinilla, Luiza Dalpiccoli Toss, and Adriano Brandelli

Supplementary Table S1

Table S1. *Bacillus velezensis* P45 susceptibility to antimicrobials by disc diffusion assay.

Antibiotic	Concentration (µg/disc)	Sensitivity [1]
Amikacin	30	S
Ampicillin	10	S
Chloramphenicol	30	S
Ciprofloxacin	5	S
Clindamycin	2	S
Erythromycin	15	S
Gentamicin	10	S
Imipenem	10	S
Levofloxacin	5	S
Linezolid	30	S
Meropemem	10	S
Norfloxacin	10	S
Penicillin	10	S
Tetracycline	30	S
Vancomycin	30	S

[1] Results are presented as susceptible (S), intermediate (I) or resistant (R).

foods MDPI

Supplementary Table S2

Table S2. Genes related to potential probiotic characteristics in *Bacillus velezensis* P45 genome.

**Gene function**	**Start**	**Stop**	**Contigs**
**Adhesion and aggregation**			
Enolase	210943	212235	2
Fibronectin-binding protein	396529	398244	1
Exopolysaccharides (EPS) biosynthesis	-		-
Glycosyl transferase family protein	175266/132838	176300/133905	2/6
Manganese-dependent protein-tyrosine phosphatase	129577	130341	12
Tyrosine-protein kinase EpsD	166183/128842	166863/129531	2/12
Tyrosine-protein kinase transmembrane modulator EpsC	165470/128109	166177/128855	2/12
Triosephosphate isomerase	208626	209387	2
Sortase D	56055	56651	1
ATP synthase α, β, γ, and δ	84097	84495	12
Flagellar hook-basal body complex FliE	342089	341769	1
**Mucin or mucus adhesion**			
Lipoprotein signal peptidase	417839	417378	1
Glyceraldehyde-3-phosphate dehydrogenase	71278	72300	7
Glutamine-binding periplasmic protein	10697	9870	13
Elongation factor Tu	28514	27324	14
Tyrosine-protein kinase transmembrane modulator EpsC	165470/128109	166177/128855	2/12
**Vitamin biosynthesis**			
Thiamine			
Xanthine/uracil/thiamine/ascorbate permease family protein	129639	130613	6

Hydroxyethylthiazole kinase	304912	305730	3
Hydroxymethylpyrimidine ABC transporter: ATPase component	20189	19407	17
Hydroxymethylpyrimidine ABC transporter: transmembrane component	19432	18620	17
Thiamin ECF transporter: substrate-specific component ThiT	14251	13670	23
Thiamin pyrophosphokinase	379675	379031	1
Thiaminase II involved in salvage of thiamin pyrimidine moiety	802404	801697	1
Riboflavin			
3,4-dihydroxy-2-butanone 4-phosphate synthase	73041	74237	10
6,7-dimethyl-8-ribityllumazine synthase	74270	74734	10
ATP phosphoribosyltransferase	114436	115077	2
Diacylglycerol kinase	41095	41475	9
Diaminohydroxyphosphoribosylaminopyrimidine deaminase/5-amino-6-(5-phosphoribosylamino) uracil reductase	71283	72398	10
NADH dehydrogenase	743449/13536	742271/12469	1/8
Orotidine 5′-phosphate decarboxylase	405975	405256	1
Phosphoribosyl-AMP cyclohydrolase/Phosphoribosyl-ATP pyrophosphatase	119062	119682	2
Riboflavin kinase/FMN adenylyltransferase	296657/283928	295716/284795	1,3
Riboflavin synthase	72379	73026	10
Ribulose-phosphate 3-epimerase	380394	379744	1
Riboflavin ECF transporter: substrate-specific component RibU	91274	91846	10
Transcription termination protein NusB	117690	118082	9
tRNA pseudouridine synthase B	296657	295716	1
Pyridoxin			
D-3-phosphoglycerate dehydrogenase	90785	89208	10
Hypothetical NagD-like phosphatase	3614	4384	8
1-deoxy-D-xylulose 5-phosphate synthase	121690	123591	9
Pyridoxal phosphate-containing protein YggS	424422	423730	1
Pyridoxal 5'-phosphate synthase (glutamine hydrolyzing), glutaminase subunit	11612	110022	22
Pyridoxal 5'-phosphate synthase (glutamine hydrolyzing), synthase subunit	12518	11634	22

Pyridoxal phosphate-dependent aminotransferase	274668	273400	5
Novel pyridoxal kinase, thiD family	332236	331424	3
NAD-dependent glyceraldehyde-3-phosphate dehydrogenase	206087	207094	2
Biotin			
3-ketoacyl-CoA thiolase/Acetyl-CoA acetyltransferase	911550	912641	1
Long-chain-fatty-acid–CoA ligase	11248	12888	1
Acetyl-CoA synthetase	47489	49207	15
Adenosylmethionine-8-amino-7-oxononanoate aminotransferase	20793	22139	1
Biotin ECF transporter: ATPase component BioM of energizing module	10091/10912	9222/10067	14/14
Biotin ECF transporter: substrate-specific component BioY 549/570 1/3 Biotin synthase	909557/32705	910114/32112	1/8
Biotin-protein ligase/ Biotin operon repressor	146101	147078	10
Competence protein F homolog, phosphoribosyltransferase domain	63835	64311	2
Biotin carboxyl carrier protein of acetyl-CoA carboxylase	115150	115629	9
Biotin carboxyl carrier protein of methylcrotonyl-CoA carboxylase	14256	14468	1
Biotin carboxylase of acetyl-CoA carboxylase	115644	116996	9
Biotin carboxylase of methylcrotonyl-CoA carboxylase	12910	14259	1
Folate			
Dihydrofolate synthase/folylpolyglutamate synthase	177155	178447	7
GTP cyclohydrolase I type 1	115527	116099	10
Pantoate-β-alanine ligase	148160	149020	10
Aspartate 1-decarboxylase	149022	149405	10
2-amino-4-hydroxy-6-hydroxymethyldihydropteridine pyrophosphokinase	3738	3235	16
Dihydroneopterin aldolase	4097	3735	16
Dihydropteroate synthase	4947	4090	16
Hypoxanthine-guanine phosphoribosyltransferase	14297	13758	16
Para-aminobenzoate synthase, amidotransferase component	6396	5809	16
5-formyltetrahydrofolate cyclo-ligase	76008	76562	9
Dihydrofolate reductase	162338/122577	162868/123083	2/4
Thymidylate synthase	121438/154824	122577/155618	4

**Aminoacids metabolism**			
Threonine			
Biosynthetic aromatic amino acid aminotransferase α	986711	985527	1
Aspartokinase	287393/41490/127813	286178/42854/129042	1/5/7
Homoserine dehydrogenase	6285	7586	8
Aspartate aminotransferase	75771/97275/153149	74461/98444/154330	5/8/10
Aspartate-semialdehyde dehydrogenase	288522	287482	1
Homoserine kinase	190284/8641	189289/9570	6/8
Threonine synthase	7577	8644	8
Tryptophan	128533	129330	10
Tryptophan synthase α chain	127338	128540	10
Tryptophan synthase β chain			
Phosphoribosylformimino-5-aminoimidazole carboxamide ribotide isomerase	117573	118310	2
Isochorismatase	38249	39175	8
Indole-3-glycerol phosphate synthase	125947	126699	10
Anthranilate phosphoribosyltransferase	124938	125954	10
Anthranilate synthase, aminase component	53934/123419	51757/124966	3/10
Methionine			
Cystathionine γ-lyase	27707	28849	13
Cystathionine γ-synthase	783786	782665	1
S-adenosylmethionine synthase	24548	25750	17
Serine acetyltransferase	48568	47915	14
Homoserine O-succinyltransferase	148015	147107	4
Methionine ABC transporter ATP-binding protein	342295	343320	2
5-methyltetrahydrofolate-homocysteine methyltransferase	14799	15935	12
5,10-methylenetetrahydrofolate reductase	862257	864098	1
O-acetylhomoserine sulfhydrylase	783786	782665	1
SAM-dependent methlytransferase YrrT	25377	26018	13
Leucine			

3-isopropylmalate dehydrogenase	55405	55551	13
2-isopropylmalate synthase	149919	151475	7
Lysine			
Aspartokinase	287393/41490/127813	286178/42854/129042	1/5/7
4-hydroxy-tetrahydrodipicolinate synthase	286148/37806	285279/37012	1/11
4-hydroxy-tetrahydrodipicolinate reductase	141845	142648	10
Aspartate-semialdehyde dehydrogenase	288522	287482	1
Diaminopimelate decarboxylase	66014	67333	10
N-acetyldiaminopimelate deacetylase	140993	139783	1
2,3,4,5-tetrahydropyridine-2,6-dicarboxylate N-acetyltransferase	592704	591994	1
N-acetyl-L,L-diaminopimelate aminotransferase	605826	606995	1
Cysteine			
Cysteine synthase	23514/8918	22543/7992	15/16
Phosphoadenylyl-sulfate reductase (thioredoxin)	404293	403592	1
Cysteinyl-tRNA synthetase	47918	46518	14
Sulfite reductase [NADPH] hemoprotein β-component	270876	272591	2
Sulfite reductase [NADPH] flavoprotein	269040	270848	2
Histidine			
Phosphoribosyl-AMP cyclohydrolase	119062	119682	2
Imidazole glycerol phosphate synthase, regulatory subunit	118307	119065	2
Imidazole-glycerol-phosphate dehydratase	116353	116937	2
Histidinol dehydrogenase	115074	116387	2
ATP phosphoribosyltransferase	113271	114443	2
Histidinol-phosphate aminotransferase	129342	130424	10
Histidinol phosphatase	13877	13065	7
Arginine			
N-acetyl-γ-glutamyl-phosphate reductase	849651	848521	1
N-acetylglutamate synthase	848498	847281	1
N-acetylglutamate kinase	847266	846490	1

N-acetylornithine aminotransferase	846493	845636	1
Ornithine carbamoyltransferase	841133	840162	1
Argininosuccinate synthase	31088	32299	7
Argininosuccinate lyase	32296	33672	7
Arginine pathway regulatory protein ArgR	124530	124979	9
**Enzyme production for food digestion**			
α-amylase	157406	155427	5
Lipases	186485	185841	5
Extracellular protease	326322	323911	3
Phytase	199030	200181	4
Beta-glucanase	74114	75613	1
Endoglucanase	29568	28069	24
Endo-1,4-beta-D-glucanase Y	213763	214494	3
Xylanase	99437	98796	12
**Stress adaptation / host gastrointestinal tract adaptation**			
Temperature tolerance			
Cold shock protein (CSP) family	1039058	1039258	1
Heat shock protein GrpE	24384	24959	9
Chaperone protein DnaJ	27008	28135	9
Chaperone protein DnaK	24984	26822	9
Heat shock protein HtpX	661806	660913	1
Ribosomal 50S subunit-recycling heat shock protein, contains S4 domain	22747	22487	16
Co-chaperonin GroES (heat shock protein)	258029	258029	6
Molecular chaperone GroEL (heat shock protein)	257987	256353	6
Acid tolerance			
ATP synthase subunit a	77973	78707	12
ATP synthase subunit b	79108	79620	12
ATP synthase subunit c	78753	78965	12
ATP synthase α chain	80179	81687	12

ATP synthase β chain	82652	84073	12
ATP synthase γ chain	81763	82626	12
ATP synthase ε chain	84097	84495	12
ATP synthase δ chain	79617	80162	12
PTS system, cellobiose-specific IIA component	261694	262026	3
PTS system, cellobiose-specific IIB component	259993	260301	3
PTS system, cellobiose-specific IIC component	260317	261675	3
ATP-dependent Clp protease ATP-binding subunit ClpC	57807	55375	14
ATP-dependent Clp protease ATP-binding subunit ClpE	639451	641547	1
ATP-dependent Clp protease ATP-binding subunit ClpX	157561	158823	7
Glucose-6-phosphate isomerase	103985	105337	8
GTP pyrophosphokinase	807871/270463	807236/271095	1/3
Pyruvate kinase	47733	49490	7
pH tolerance			
Na+/H+ antiporter NhaC	78141	76882	9
K+/H+ antiporter YhaU	95822	959439	1
Ca2+/H+ antiporter	70389	69337	6
Alkaline shock proteins	117018	117425	9
F0F1-ATPase	77582	77965	12
Bile salts tolerance			
Glucosamine-6-phosphate deaminase	107970	107245	2
CTP synthase	53504	55111	12
Osmotic stress tolerance			
Glycine betaine	13128	11593	15
ABC transporter, ATP-binding protein OpuAA	163928	162672	5
Glycine betaine ABC transport system, glycine betaine-binding protein OpuAC	161823	160942	5
ABC transporter, substrate binding protein and permease	10107	8830	15
ABC transporter, binding protein and permease	11446	10718	13
Oxidative stress tolerance			

Catalase KatE	1062973	1064418	1
Thiol peroxidase	2773	2300	6
Glutathione reductase	339677	340033	2
Glutathione peroxidase	148295	148777	4
NADH flavin oxidoreductase	171787	170771	9
Iron dependent peroxidase	306815	308071	3
Thioredoxin reductase	127725/121949/25498/100074	128678/120939/24503/101057	2/5/8/10
Cadmium-/manganese-transporting P-type ATPase	261405	263516	2

foods MDPI

Supplementary Figure S1

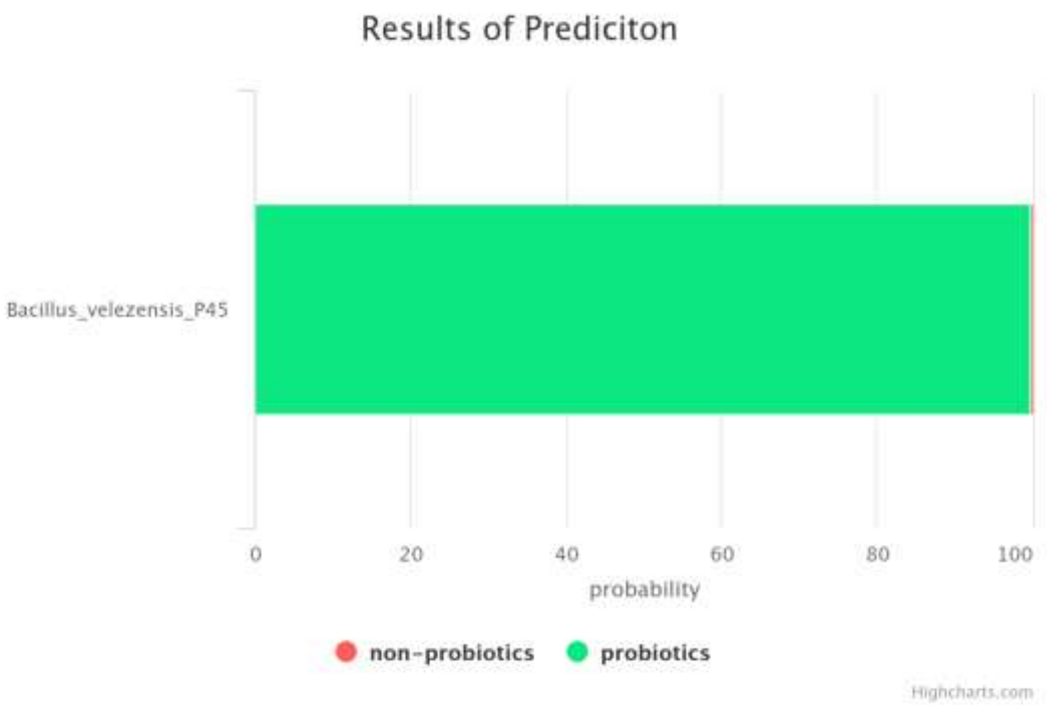

Figure S1. Probability prediction of *B. velezensis* P45 genome as probiotics using the iProbiotics algorithm. The probiotic score was 0.9988118327 in a scale from 0 to 1.

Supplementary Figure S2

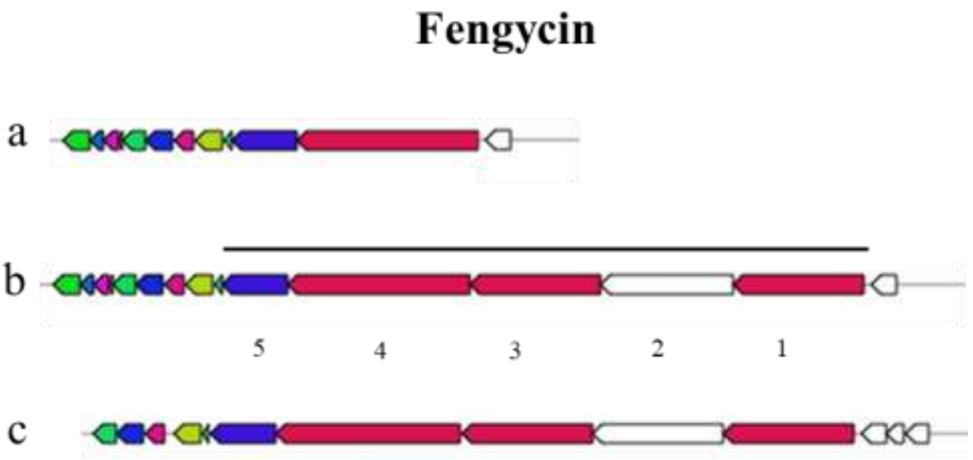

Figure S2. Biosynthetic gene cluster for fengycin of the query sequence *Bacillus velezensis* P45. Comparison of P45 cluster (**a**) with reference *B. velezensis* FZB42 clusters of (**b**) fengycin and (**c**) plipastatin. The highlighted core biosynthetic genes are represented by (1*) fenA*, (2) *fenB*, (3) *fenC*, (4) *fenD*, (5) *fenE*.

Supplementary Figure S3

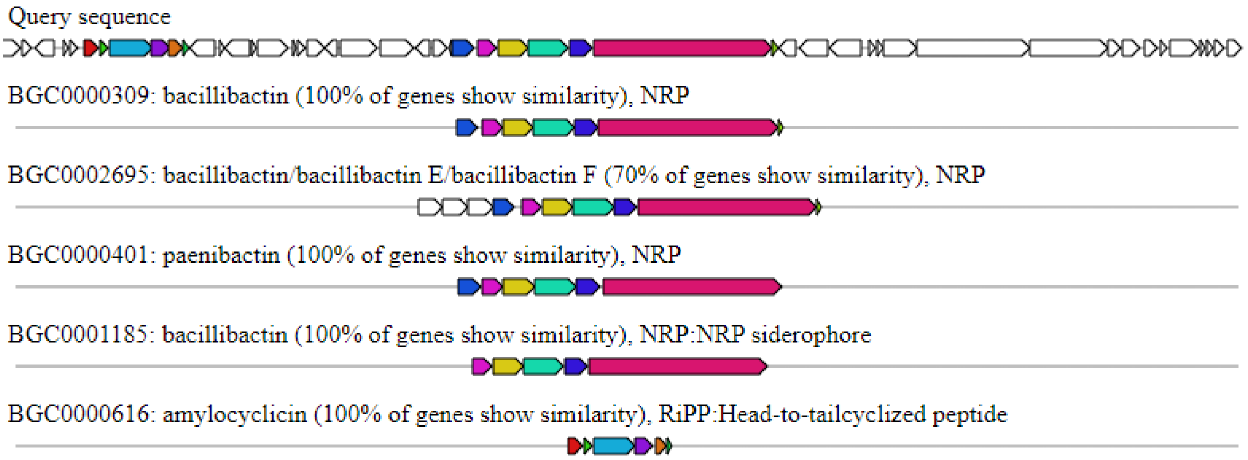

Figure S3. Bacillibactin gene cluster of the query sequence *Bacillus velezensis* P45 identified by antiSMASH 7.0.

Supplementary Table S3

Table S3. Predicted bacterial RiPP clusters in *Bacillus velezensis* P45 genome based on BAGEL4.

AOI	Start	End	Class
NZ_JAFJZY0100000061.21.AOI_01	56597	76597	Sactipeptides
NZ_JAFJZY0100000081.24.AOI_01	9524	29857	266.1; Amylocyclicin
NZ_JAFJZY0100000081.24.AOI_02	59861	80023	320.1; ComX3
NZ_JAFJZY0100000081.24.AOI_03	181820	201381	225.2; UviB
NZ_JAFJZY0100000051.8.AOI_01	122972	143107	132.2; LCI
NZ_JAFJZY0100000051.8.AOI_02	133319	153319	LAPs/Thiopeptide

Supplementary Table S4

Table S4. Predicted secondary metabolites gene clusters in *Bacillus velezensis* P45 genome based on PRISM4.

**Clusters**	**Cluster type**
Cluster 1	Nonribosomal peptide
Cluster 2	Class II/III confident bacteriocin (thiopeptide)
Cluster 3	Nonribosomal peptide
Cluster 4	Polyketide/ Nonribosomal peptide
Cluster 5	Polyketide/ Nonribosomal peptide
Cluster 6	Polyketide
Cluster 7	Nonribosomal peptide
Cluster 8	Polyketide
Cluster 9	Bacterial head-to-tail cyclized peptide
Cluster 10	Nonribosomal peptide
Cluster 11	ComX
Cluster 12	Nonribosomal peptide
